# Spontaneous hemorrhage from splenic tissue 13 years after total splenectomy: report of a case

**DOI:** 10.1186/s40792-015-0099-0

**Published:** 2015-10-05

**Authors:** Takehiro Maki, Makoto Omi, Daisuke Ishii, Hiroyuki Kaneko, Kenjiro Misu, Hitoshi Inomata, Masatoshi Tateno, Kazuyoshi Nihei

**Affiliations:** Department of Surgery, Kushiro Red Cross Hospital, 21-14, Shineichyo, Kushiro, Hokkaido 085-8512 Japan; Department of Pathology, Kushiro Red Cross Hospital, 21-14, Shineichyo, Kushiro, Hokkaido 085-8512 Japan

**Keywords:** Hemorrhage, Rupture, Accessory spleen, Splenosis, Splenectomy

## Abstract

A 33-year-old man suffered sudden abdominal distension without traumatic episodes. He had undergone total splenectomy for hereditary spherocytosis 13 years ago. He was in shock, and his hemoglobin level was 10.5 g/dl. Contrast enhanced computed tomography revealed a giant mass in the left upper abdomen and extravasation of the contrast material into the mass. Excision of the mass was performed, and microscopic examination showed a giant hematoma surrounded by normal splenic tissue. We speculated that an accessory spleen or splenosis had enlarged for the 13 years and ruptured. The patient remained asymptomatic 4 months after the surgery. Spontaneous hemorrhage from accessory spleens or splenosis is extremely rare, and relevant case reports suggest that surgical resection of bleeding sites yields favorable prognosis although preoperative qualitative diagnosis seems to be difficult.

## Background

We often encounter accessory spleens in radiological studies, abdominal surgeries, or autopsies. Splenosis is also well known as autotransplantation of splenic tissue caused by splenic trauma or splenectomy. These kinds of ectopic splenic tissue are usually associated with no symptoms. We here present a very rare case which suffered life-threatening bleeding from splenic tissue without traumatic episodes although total splenectomy had been done 13 years ago, followed by a review of the literature.

## Case presentation

**Fig. 1:**
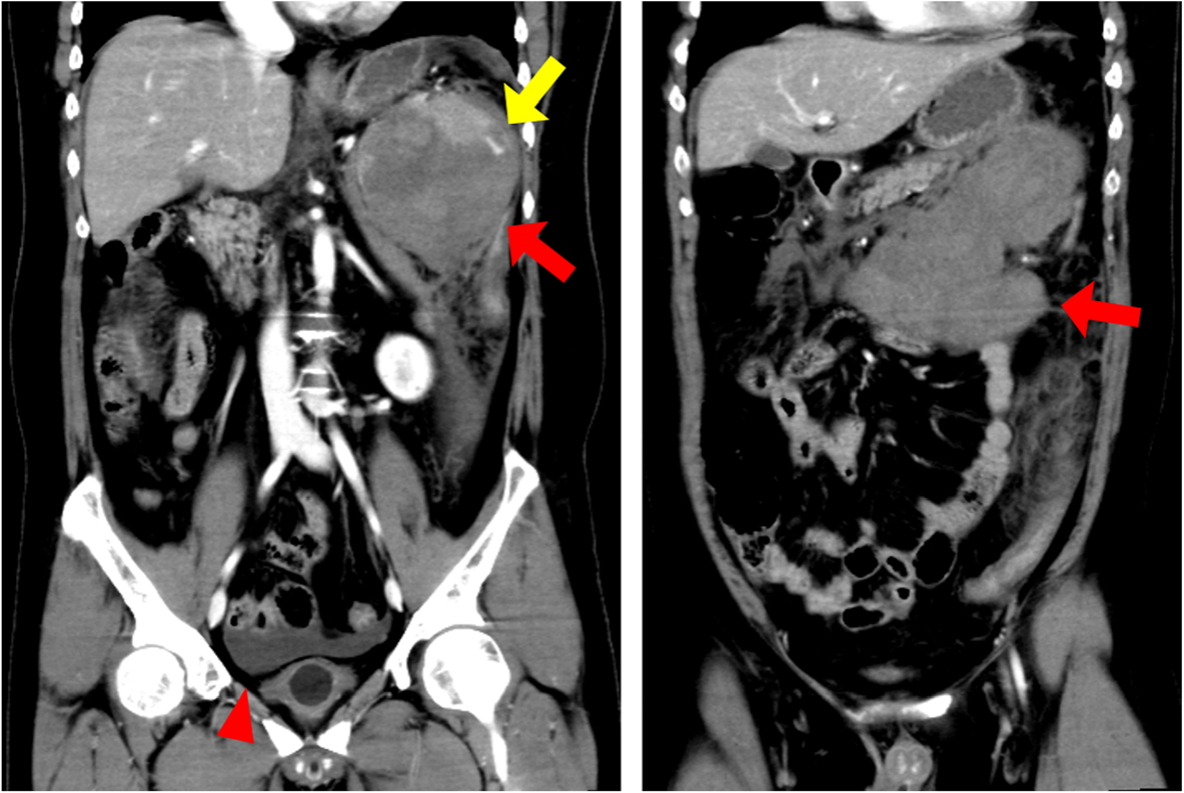
Images of frontal sections of contrast-enhanced computed tomography at a portal phase. *Red arrows* show heterogeneously enhanced abdominal mass which ranges from an intramesenteric space of the transverse colon to the left upper abdomen with extension to 25 cm in the longest diameter. A *yellow arrow* shows extravasation of the contrast material into the mass. An *arrowhead* shows ascites in Douglas’ pouch

A 33-year-old Japanese man suffered sudden abdominal distension and visited our hospital by ambulance. He did not have any symptoms before the onset and denied histories of trauma. He had undergone cardiac surgery for arterial septal defect at 0 year old and total splenectomy for hereditary spherocytosis at 20 years old. The splenectomy was performed at another hospital, and the patient had not been clinically followed up since the surgery. He was 162-cm tall and weighted 75 kg (body mass index, 28.6). His blood pressure, pulse, oxygen saturation, and body temperature were 70/42 mmHg, 102 beats/min, 99 %, and 37.0 °C, respectively. His abdomen was considerably distended, and tenderness was observed at the left side of the abdomen. Laboratory examinations revealed intense acute inflammation (leukocyte count, 42,710/μl; C-reactive protein level, 0.80 mg/dl), anemia (hemoglobin level, 10.5 g/dl), mild liver dysfunction (total bilirubin, 3.03 mg/dl; direct bilirubin, 0.84 mg/dl), elevated levels of pancreatic enzymes (amylase, 146 IU/l; lipase, 240 U/l), and mild renal dysfunction (creatinine, 1.51 mg/dl; blood urea nitrogen, 15.4 mg/dl). Contrast-enhanced computed tomography revealed a heterogeneously enhanced abdominal mass with extension to 25 cm in the longest diameter; it ranged from an intramesenteric space of the transverse colon to the left upper quadrant of the abdomen in which the primary spleen would exist if splenectomy had not been performed. Ascites in Douglas’ pouch and extravasation of the contrast material into the mass in a portal phase were also observed (Fig. 1). We diagnosed hemorrhagic shock due to bleeding from some kind of giant neoplasm of the abdomen such as gastrointestinal stromal tumor.

**Fig. 2:**
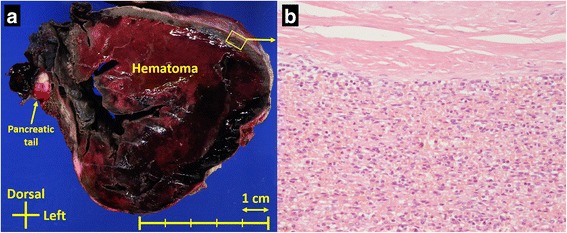
Findings of an excised specimen. **a** On gross examination, the excised specimen with a diameter of 9 cm shows a giant hematoma surrounded by gray hard parenchyma with a smooth capsule. **b** Microscopically, the specimen showed normal splenic tissue with a firm capsule

We performed urgent laparotomy to restrain the bleeding. Bloody ascites was observed. A great amount of hematoma was contained in the mesentery of the transverse colon and removed. Connected to the intramesenteric hematoma, a giant mass was observed at the left upper quadrant of the abdomen. It looked like just a hematoma or some kind of neoplasm and was firmly adhered to the pancreatic tail; it was excised with the pancreatic tail. The intraoperative hemorrhage volume was 5943 g, and 1820 ml of concentrated blood cells and 480 ml of fresh frozen plasma were transfused. On gross examination, the excised specimen showed a giant hematoma surrounded by gray hard parenchyma with a smooth capsule. Microscopic examination of the specimen revealed that the parenchyma was pathologically normal splenic tissue (Fig. 2).

We speculated that an accessory spleen which had survived the past splenectomy or splenosis which had spread to the abdomen at the splenectomy grew to a large size and ruptured for any reasons. After the surgery, the patient suffered pancreatic fistula which corresponded to grade B in the International Study Group of Pancreatic Fistula classification [1], recovered with percutaneous drainage, and was discharged 2 months after the surgery. Postoperative bilirubin level remained high and decreased to the normal level 4 weeks after the surgery (total bilirubin, 0.72 mg/dl; indirect bilirubin, 0.56 mg/dl). He remained asymptomatic 4 months after the surgery.

### Discussion

Accessory spleens occur due to the failure of the normal fusion of multiple nodules of splenic tissue originating in the dorsal mesogastrium during embryonic life [2]. They have all the histologic components of a normal spleen such as the hilum, white pulp, and capsule and receive their blood supply from a branch of the splenic artery [3]. Accessory spleens are observed in 11–33 % patients on necropsies [2] and are usually single or rarely exceed six in number [4]. Common locations of accessory spleens are in the splenic hilum (75 %) and in the tail of the pancreas (25 %), although they have also been found in other locations, such as the scrotum, the right iliac fossa, and the retroperitoneal space [5, 6]. Most of accessory spleens do not cause any symptoms and accidentally discovered by imaging tests or laparotomies for other purposes, but complications such as hemorrhage or rupture which are well recognized in the primary spleen can also occur in the accessory spleens [7].

Splenosis means autotransplantation of splenic tissue; cells from the pulp of the damaged spleen spill out and grow as nodules of the splenic tissue [3, 4]. Therefore, it occurs after splenic injury or splenectomy. Histopathologically compared to the primary spleen and accessory spleens, splenic implants have a thinner capsule which is devoid of elastic tissue and the white pulp area or hilum is usually deficient [8]. Differently from accessory spleens, splenic implants receive their blood supply from the surrounding tissue [9]. The frequency of splenosis due to splenectomy after traumatic rupture was reported to be 67 % [10]. The number of such implants varies from a single to hundreds, and their size ranges from 1 to 12 cm with an average of 3 cm [3]. Splenosis can generate anywhere, for example, peritoneum, retroperitoneum, liver, walls of any digestive tracts, gynecologic organs, subcutaneous tissue, and thorax [11]. Splenosis is mostly harmless for patients but can cause acute abdominal pain, intraabdominal hemorrhage, bowel obstruction, and gastrointestinal hemorrhage due to bowel involvement [12].

Accessory spleens and splenosis are distinguished from those microscopic findings but some researchers insist that the distinction is actually difficult [3, 13]. In the present case, the excised splenic tissue had a firm capsule but did not present apparent hilum or white pulp area, and thus, we could not precisely diagnose as an accessory spleen or splenosis. At any rate, it is obvious that some kind of hypertrophy of splenic tissue occurred for 13 years after total splenectomy. As Beahrs et al. described [14], this enlargement of splenic tissue might have been compensatory hypertrophy for splenectomy. If so, we should take care of the recurrence of symptomatic hypertrophy of the residual splenic cells which were strewn all over the abdomen by the rupture. On the other hand, this case presented intense acute inflammation of unknown cause; some kind of strong infection or immunological response might have triggered the atraumatic rupture of the exaggerated splenic tissue. In patients who underwent splenectomy because of a chronic hemolytic disorder or immune thrombocytopenia, ectopic splenic tissue such as splenosis can result in recurrence of them [15]. Our case presented the elevation of indirect bilirubin which may indicate recurrence of hereditary spherocytosis due to the enlargement of the splenic tissue. If the present case was followed up, periodical examinations would indicate resection of the remnant splenic tissue. While risk factors of bleeding or rupture of accessory spleen or splenosis are not revealed, rapid enlargement of the splenic tissue may indicate its excision. Moreover, we must be careful for overwhelming post-splenectomy infection. Although it is relatively rare and the clinical management is not well established, it has a high mortality rate with delayed or inadequate treatment [16]. The most critical action in the management of overwhelming post-splenectomy infection is vigilance against *Streptococcus pneumoniae* and the immediate use of broad-spectrum intravenous antibiotics, ideally based on the result of blood cultures [17].

Spontaneous bleeding from accessory spleens and splenosis is extremely rare, and only 18 cases have been reported including this case [11, 18–33] (Table 1). The English language literature was extracted from PubMed from 1970 to 2015 using the following Medical Subject Heading terms: “hemorrhage” or “spontaneous rupture” in combination with “accessory spleen” or “splenosis”. Age of onset ranged from 11 to 65 and its median was 43. Male to female ratio of the patients was 2:1. Eleven patients (61 %) complained of local pain which was the most common symptom. Fifteen patients (83 %) had undergone splenectomy; the duration between splenectomy and bleeding ranged from 2 to 41 years, and its median was 19 years. Six patients (33 %) suffered hemorrhagic shock. Perioperative qualitative diagnosis of bleeding mass was properly made using computed tomography, magnetic resonance imaging, ^99m^Tc sulfur colloid study, or fine-needle aspiration cytology in six cases [19, 20, 23–25, 29] (33 %) while excised specimens eventually revealed splenic tissue in the other 12 cases (66 %). Preoperative angiography was performed in four cases [19, 23, 26, 28] and effective to detect bleeding sites in two cases [23, 28]. Four cases (22 %) were finally diagnosed as accessory spleens while 12 cases (66 %) were diagnosed as splenosis. Size of bleeding mass varied and ranged from 1 to 11 cm. Locations of splenic tissue and hemorrhagic space also varied: in seven cases (39 %), splenic tissue involved walls of intestines and caused gastrointestinal hemorrhage; and in six cases (33 %), splenic tissue occurred in the left upper abdomen and caused intraperitoneal or retroperitoneal hemorrhage. As treatments for the bleeding, surgical resection of the splenic tissue was performed in 16 cases (89 %) while 2 cases with mild hemorrhage (11 %) did not need any intervention. Angiography for hemostasis was not performed in any 18 cases. Clinical outcomes were generally favorable. In the present case, preoperative vital signs and results of various examinations indicate hemorrhagic shock due to the mass in the left upper abdomen but the precise cause was revealed by postoperative microscopic findings. In summary of the 18 cases including the present case, excision of bleeding sites leads to favorable prognosis for spontaneous bleeding or rupture from accessory spleens or splenosis although preoperative qualitative diagnosis seems to be difficult.

**Table 1:** Reported 18 cases of spontaneous bleeding from accessory spleens or splenosis

Reference	Age/sex	Major complaint	History of splenectomy (cause and age of splenectomy)	Hemorrhagic shock	Bleeding mass			Hemorrhagic space	Treatment	Outcome
					Sort	Size (cm)	Location			
1974, Texeira [18]	11/F	Pain, pyrexia, vomiting	−	−	AS	2	LUA	Intraperitoneal	Excision	7 days, discharged
1989, Basile [19]	24/M	Pain, melena	+ (trauma, 5 years old)	+	SP	1–5	Ileum	Gastrointestinal	Excision	7 days, discharged
1990, Goodman [20]	36/M	Pain, fatigue, pyrexia, anorexia	+ (trauma, 6 years old)	−	AS	ND	LUA	Subcapsule of the AS	Excision	ND
1991, Cuckow [21]	44/M	Pain	+ (trauma, 32 years old)	−	SP	2	Duodenum	Intraperitoneal	Excision	Recovered
1991, Feferman [22]	31/F	Pain	+ (trauma, 9 years old)	+	SP	5–6	Uterine ligament	Intraperitoneal	Excision	Recovered
1992, Cordier [23]	50/M	Hemoptysis, pain	+ (trauma, 22 years old)	−	SP	ND	Left pleura	Intrapulmonary	Excision	11 months, asymptomatic
1996, Chiarugi [24]	65/M	Hematemesis, melena	+ (Gaucher’s disease, 36 years old)	+	SP	11	Stomach	Gastrointestinal	Excision	9 days, discharged
1998, Katz [25]	65/F	Pain, nausea	+ (trauma, 45 years old)	−	SP	3	LUA	Retroperitoneal	Observation	Several months, asymptomatic
1999, Coote [26]	50/F	Pain, vomiting, malaise	−	−	AS	5	LUA	Intraperitoneal	Excision	4 days, discharged
1999, Padilla [27]	29/M	Pain, vomiting	−	+	AS	ND	LUA	Intraperitoneal	Excision	No complications
2000, Sikov [28]	48/M	Fatigue, melena	+, (trauma, 7 years old)	+	SP	ND	Small bowel	Gastrointestinal	Excision	Several years, asymptomatic
2001, Syed [29]	49/M	Hemoptysis	+ (trauma, several years ago)	−	SP	4	Left pleura	Intrapulmonary	Observation	ND
2008, Margari [30]	47/M	Gastrointestinal bleeding	+ (trauma, 28 years old)	ND	SP	5	Stomach	Gastrointestinal	Excision	ND
2009, Depypere [31]	62/F	Pain	+ (trauma, 49 years old)	−	ID	4.5	LUA	Retroperitoneal	Excision	Recovered
2012, Obokhare [32]	41/M	Pain, constipation, melena	+ (gastric varices, 39 years old)	−	SP	6.5	Colon	Gastrointestinal	Excision	Recovered
2013, Hiranyatheb [11]	16/F	Hematemesis	+ (thalassemia, 5 years old)	−	SP	4	Stomach	Gastrointestinal	Excision	3 years, asymptomatic
2013, Yang [33]	42/M	Melena	+ (trauma, 25 years old)	−	SP	5	Stomach	Gastrointestinal	Excision	Recovered
2015, Maki [The present case]	33/M	Abdominal distension	+ (spherocytosis, 20 years old)	+	ID	9	LUA	Intraperitoneal, intramesenteric, and retroperitoneal	Excision	4 months, asymptomatic

*ND* not described, *AS* accessory spleen, *SP* splenosis, *ID* indeterminate, *LUA* left upper abdomen

## Conclusions

We experience a case of spontaneous hemorrhage from splenic tissue 13 years after total splenectomy. The tissue is considered to be an accessory spleen or splenosis which had enlarged for the 13 years and ruptured. Spontaneous hemorrhage from accessory spleens or splenosis is extremely rare, and relevant case reports suggest that surgical resection of bleeding sites yields favorable prognosis although preoperative qualitative diagnosis seems to be difficult.

## Consent

Written informed consent was obtained from the patient for publication of this case report and any accompanying images. A copy of the written consent is available for review by the Editor-in-Chief of this journal.

## Abbreviations

AS: accessory spleen
ID: indeterminate
LUA: left upper abdomen
ND: not described
SP: splenosis

## References

[1] Bassi C, Dervenis C, Butturini G, Fingerhut A, Yeo C, Izbicki J, Neoptolemos J, Sarr M, Traverso W, Buchler M, International Study Group on Pancreatic Fistula Definition (2005). Postoperative pancreatic fistula: an international study group (ISGPF) definition. Surgery.

[2] Santamaría G, Vilana R, Salvadó E, Clavero JA, Ayuso MC, Luburich P (1992). Accessory spleen: ultrasonographic and tomographic characteristics. Rev Esp Enferm Dig..

[3] Case records of the Massachusetts General Hospital. Weekly clinicopathological exercises. Case 29–1995. A 65-year-old man with mediastinal Hodgkin’s disease and a pelvic mass. N Engl J Med. 1995; 333: 784–91. 10.1056/NEJM199509213331208 7643887

[4] Fleming CR, Dickson ER, Harrison EG (1976). Splenosis: autotransplantation of splenic tissue. Am J Med..

[5] Urruchi Fernández P, Cavero Rebollo O, Liedana Torres JM, Rodríguez Vela L, Martínez Bengoechea J, Gil Sanz J, Rioja Sanz LA (1993). Supernumerary spleen. Arch Esp Urol..

[6] Chateil JF, Arboucalot F, Perel Y, Roy D, Vergnes P, Diard F (1996). Acute torsion of an accessory spleen. J Radiol..

[7] Halpert B, Gyorkey F (1959). Lesions observed in accessory spleens of 311 patients. Am J Clin Pathol..

[8] Stovall TG, Ling FW (1988). Splenosis: report of a case and review of the literature. Obstet Gynecol Surv..

[9] Bock DB, King BF, Hezmall HP, Oesterling JE (1991). Splenosis presenting as a left renal mass indistinguishable from renal cell carcinoma. J Urol..

[10] Livingston CD, Levine BA, Lecklitner ML, Sirinek KR (1983). Incidence and function of residual splenic tissue following splenectomy for trauma in adults. Arch Surg..

[11] Hiranyatheb P, Euanorasetr C, Suwanthanma W, Supsamutchai C (2013). Upper gastrointestinal bleeding from gastric splenosis; A case report and literature review. J Med Assoc Thai..

[12] Sirinek KR, Livingston CD, Bova JG, Levine BA (1984). Bowel obstruction due to infarcted splenosis. South Med J..

[13] Carr NJ, Turk EP (1992). The histological features of splenosis. Histopathology..

[14] Beahrs JR, Stephens DH (1980). Enlarged accessory spleens: CT appearance in postsplenectomy patients. AJR Am J Roentgenol..

[15] Varughese N, Duong A, Emre S, Xu M, Lee A (2013). Clinical problem-solving. Venting the spleen. N Engl J Med.

[16] Sinwar PD (2014). Overwhelming post splenectomy infection syndrome—review study. Int J Surg..

[17] Morgan TL, Tomich EB (2012). Overwhelming post-splenectomy infection (OPSI): a case report and review of the literature. J Emerg Med..

[18] Texeira MB, Hardin WJ (1974). Spontaneous rupture of accessory spleen. Am Surg..

[19] Basile RM, Morales JM, Zupanec R (1989). Splenosis. A cause of massive gastrointestinal hemorrhage. Arch Surg.

[20] Goodman P, Raval B, King FA (1990). Spontaneous necrosis and hemorrhage in an enlarged accessory spleen: CT demonstration. Comput Med Imaging Graph..

[21] Cuckow P (1991). Spontaneous rupture: a new complication of splenosis. J R Coll Surg Edinb..

[22] Feferman I, Cramer J (1991). Splenosis: an unusual cause of intraabdominal hemorrhage. J Emerg Med..

[23] Cordier JF, Gamondes JP, Marx P, Heinen I, Loire R (1992). Thoracic splenosis presenting with hemoptysis. Chest..

[24] Chiarugi M, Martino MC, Buccianti P, Goletti O (1996). Bleeding gastric ulcer complicating splenosis in type 1 Gaucher’s disease. Eur J Surg..

[25] Katz DS, Moshiri M, Smith G, Meiner EM, Fruauff AA (1998). Spontaneous hemorrhage of abdominal splenosis. J Comput Assist Tomogr..

[26] Coote JM, Eyers PS, Walker A, Wells IP (1999). Intra-abdominal bleeding caused by spontaneous rupture of an accessory spleen: the CT findings. Clin Radiol..

[27] Padilla D, Ramia JM, Martin J, Pardo R, Cubo T, Hernandez-Calvo J (1999). Acute abdomen due to spontaneous torsion of an accessory spleen. Am J Emerg Med..

[28] Sikov WM, Schiffman FJ, Weaver M, Dyckman J, Shulman R, Torgan P (2000). Splenosis presenting as occult gastrointestinal bleeding. Am J Hematol..

[29] Syed S, Zaharopoulos P (2001). Thoracic splenosis diagnosed by fine-needle aspiration cytology: a case report. Diagn Cytopathol..

[30] Margari A, Amoruso M, D’Abbicco D, Notarnicola A, Epifania B (2008). Massive gastrointestinal bleeding due to splenotic nodule of the gastric wall. A case report. Chir Ital.

[31] Depypere L, Goethals M, Janssen A, Olivier F (2009). Traumatic rupture of splenic tissue 13 years after splenectomy. A case report. Acta Chir Belg.

[32] Obokhare ID, Beckman E, Beck DE, Whitlow CB, Margolin DA (2012). Intramural colonic splenosis: a rare case of lower gastrointestinal bleeding. J Gastrointest Surg..

[33] Yang K, Chen XZ, Liu J, Wu B, Chen XL, Hu JK. Splenosis in gastric wall mimicking gastrointestinal stromal tumor. Endoscopy. 2013; 45 Suppl 2 UCTN: E82-3. 10.1055/s-0032-1326263 23526531

